# Analysis of spatiotemporal characteristics and influencing factors of residents’ physical activity in urban green spaces

**DOI:** 10.3389/fpubh.2026.1710326

**Published:** 2026-05-20

**Authors:** Xishan Liu, Junhong Chao

**Affiliations:** 1 School of Physical Education, Nanyang Normal University, Nanyang, Henan, China; 2 School Hospital, Nanyang Normal University, Nanyang, Henan, China

**Keywords:** perceived environment, physical activity, questionnaire survey, spatiotemporal characteristics, urban green space, urban parks

## Abstract

**Introduction:**

Urban green spaces are important settings for residents’ physical activity (PA), yet evidence remains limited on how park-based PA varies across time and space and how perceived environmental conditions are associated with PA-related outcomes. This study examines the spatiotemporal characteristics of PA in six typical urban parks in Zhengzhou, China, using a multi-source dataset.

**Methods:**

Data were collected through systematic field observations (18,650 valid activity records, geo-tagged via GPS), questionnaires (345 valid responses), and Volunteered Geographic Information (VGI) comprising OpenStreetMap-sourced park boundary and facility data. These two data sources serve complementary but analytically distinct purposes: the observational and VGI data are used to document spatiotemporal patterns of park-based PA, while the questionnaire data provide the individual-level basis for examining associations between perceived environmental conditions and self-reported PA outcomes. GIS-based spatial analysis was used to identify activity hotspots and compare temporal and group-specific patterns. Questionnaire-based individual-level analyses were used to examine associations between perceived environmental conditions and PA-related outcomes.

**Results:**

Clear spatiotemporal differences were observed in park-based PA. Activity frequency was higher on weekends than on weekdays, middle-aged and older adults represented the largest share of observed park users, and activity peaked mainly during midday and evening periods. Spatially, PA hotspots were concentrated in leisure, fitness, and recreation zones, with differences across gender, age group, and activity intensity. The questionnaire-based individual-level analyses suggested that perceived safety, facility convenience, and environmental quality were positively associated with PA-related outcomes, whereas noise and crowding tended to show negative associations.

**Conclusion:**

Taken together, the spatiotemporal and individual-level findings indicate that park-based PA in Zhengzhou is associated with both temporal rhythms tied to daily work-rest cycles and perceived environmental conditions. Facility provision, perceived safety, and the management of noise and crowding emerge as particularly relevant factors for sustaining park use. These context-specific findings have practical implications for park design and management, though broader generalization requires research across more diverse urban settings.

## 1 Introduction

As the pace of global urbanization continues to accelerate, urban green spaces have gradually become indispensable public resources in residents’ daily lives (1). Green spaces not only fulfill vital ecological functions such as improving air quality, regulating climate, and maintaining ecological balance, but also serve as primary venues for recreation, social interaction, and exercise at the societal level (2). Extensive research indicates that rational utilization of green spaces can effectively promote PA among residents, thereby enhancing physical and mental health, reducing chronic disease incidence, and improving overall urban livability and public health standards (3). However, despite widespread recognition of the health benefits of urban green spaces, significant disparities persist in their actual utilization across different cities and populations. Their spatiotemporal distribution characteristics and associated contextual factors require further in-depth investigation.

In recent years, accelerated lifestyles and heightened health awareness have driven trends toward diversified and regularized PA patterns (4). As a representative setting for PA, green space utilization may be associated not only with objective environmental factors such as spatial distribution and facility conditions but also with residents’ subjective perceptions and behavioral preferences. From a theoretical perspective, the Social Ecological Model (SEM) and environmental psychology theories provide useful interpretive lenses for understanding how environmental conditions shape park-based physical activity. In this study, these frameworks are used to interpret spatiotemporal activity patterns and questionnaire-based individual-level associations involving perceived environmental conditions. For instance, factors such as green space size, accessibility, internal pathway layout, and lighting facilities have often been reported as correlates of the frequency and duration of residents’ PA participation (5). According to environmental psychology theories, particularly stress–restoration and perceived safety frameworks, these physical attributes may matter not only through functional availability but also through collective perceptions of comfort, security, and environmental stress, which are relevant to sustained physical activity engagement. Concurrently, different demographics exhibit variations in temporal preferences and spatial choices. Middle-aged and older adult individuals tend to engage in activities during morning and evening hours, while adolescents and working professionals are more constrained by work and academic schedules (6). Moreover, the spatial distribution disparities among light, moderate, and vigorous physical activities reflect the interactive relationship between green space functional configurations and population needs (7).

Regarding research methodologies, traditional questionnaires and field observations effectively reveal basic characteristics of residents’ PA but face limitations in spatiotemporal coverage and data granularity (8). With the proliferation of information technology and widespread adoption of fitness apps, running and exercise data derived from VGI offer new avenues for PA research (9). Such data not only precisely capture residents’ movement patterns, time preferences, and activity intensity but also, when combined with GIS analysis, provide deeper insights into the complex relationship between PA and green space environments. Therefore, integrating multi-source data has become a key development direction in current research on urban green spaces and residents’ PA (10).

Based on this background, this study aims to examine park-based PA in six typical urban parks in Zhengzhou through two complementary analytical tracks. The first track uses observational and GIS/VGI data to document the spatiotemporal characteristics of PA, including temporal variation across time periods and user groups and spatial clustering across park zones. The second track uses questionnaire-based individual-level analyses to examine associations between perceived environmental conditions and PA-related outcomes among park users. These two tracks address different research questions and rely on different data sources; they are not combined into a single integrated model but are presented together to provide a more complete empirical picture of park-based PA.

This study makes two contributions. First, it provides a multi-source empirical description of the temporal and spatial characteristics of park-based PA in a mid-sized Chinese city, thereby extending context-specific evidence on how activity patterns vary across time periods, park zones, and user groups. Second, it uses questionnaire-based individual-level evidence to examine how perceived environmental conditions, including safety, facility convenience, crowding, and noise, are associated with PA-related outcomes.

## 2 Related work

### 2.1 Urban green spaces and resident health

Urban green spaces constitute a vital component of modern urban ecosystems, playing an irreplaceable role in enhancing environmental quality and promoting public health. Extensive research indicates that green spaces create comfortable environments for exercise and recreation by regulating microclimates (11), purifying air (12), reducing noise (13), and improving landscape quality (14). A well-maintained green environment not only motivates residents to engage in physical activities (15) but also effectively alleviates stress (16), enhancing wellbeing and psychological recovery. Furthermore, the accessibility and equity of green spaces are critical topics in public health research. Scholars have found that parks and green areas with rational spatial distribution can effectively narrow health disparities among different social groups (17), advancing the goal of “healthy cities.” Existing research indicates that the health benefits of green spaces depend on whether residents actually use them (18). Currently, green spaces vary across regions in distribution and environmental characteristics. Therefore, identifying which features encourage more active use of green spaces is crucial. This not only helps understand the practical benefits of green spaces but also provides clear directions for optimizing them. Ultimately, this enables green spaces to more effectively promote public health and wellbeing.

### 2.2 Relationship between green spaces and PA

With the advancement of the Healthy China strategy, the relationship between residents’ PA and green space utilization has emerged as a research focus. Existing studies reveal that the size, form, and connectivity of green spaces, along with the surrounding built environment, significantly influence residents’ PA levels. For instance, Li et al.’s (18) empirical research indicates that large parks are more likely to attract residents for moderate-to-vigorous activities, while small neighborhood green spaces primarily support light activities. Liu et al. (19) noted that surrounding population density, public transit accessibility, and service facility availability significantly influence the frequency and duration of residents’ PA. Fan et al. (20), using a combination of questionnaires and observation, found pronounced gender and age differences in activity selection: middle-aged and older adult residents tended to walk or participate in square dancing in the early morning, while younger residents preferred evening jogging or vigorous-intensity PA. Bianconi et al. (21) empirically demonstrated that vegetation coverage and accessibility of urban green spaces positively influence residents’ walking and exercise levels, thereby reducing cardiovascular disease risk. He et al. (22) proposed that green spaces exhibit significant spatiotemporal connectivity, particularly in high-density urban settings, where park characteristics like tree coverage and facility provision enhance PA diversity. Furthermore, Li et al. (23) explored the correlation between green space quality and PA, finding that landscape diversity and lighting facilities positively promote moderate activity participation. Bao et al. (24) examined the relationship between urban green space characteristics and PA levels, discovering that elements such as tree coverage, exercise equipment, and picnic areas significantly enhance activity intensity. Stangierska et al. (25) found through surveys that increased residential greening rates significantly raised residents’ frequency of leisure walking and jogging. Heo and Bell (26) analyzed the impact of spatiotemporal inequities in green space on health equity, noting that insufficient green space in low-income communities leads to lower PA levels. Taken together, these studies from China, Europe, and North America (21, 25, 26) suggest that accessibility, amenities, vegetation, and perceived environmental quality are recurring correlates of PA, but the magnitude and even direction of observed relationships can vary across urban contexts, populations, and analytical scales.

### 2.3 Evolution of data and methods

Traditionally, researchers primarily relied on questionnaires and field observations to gather resident activity data. While these methods provide intuitive insights into behavioral patterns, they face limitations in sample coverage, spatiotemporal resolution, and objectivity. In recent years, with advancements in information technology, big data-driven research methodologies have gained prominence. Behavioral big data (e.g., transit card swipes, social media check-ins, mobile phone signaling) has been widely applied in green space utilization studies (27). For instance, Lou et al. (28) used big data to reveal spatiotemporal variations in urban green space vitality, emphasizing the influence of season and time period on activity distribution. Park et al. (29) analyzed spatiotemporal patterns of urban green space use based on mobile phone signaling data, highlighting the impact of external factors like transportation accessibility on activity frequency.

Concurrently, the proliferation of fitness apps and wearable devices has opened new avenues for PA research. The vast volumes of VGI data generated by fitness software feature high trajectory accuracy and strong temporal continuity, authentically reflecting residents’ exercise timing, routes, and intensity. Shi and Gao (30) analyzed fitness app data to study the spatiotemporal distribution of residents’ running activities, revealing distinct morning and evening peaks, with waterfront greenways and large parks emerging as activity hotspots. Wei et al. (31) employed geographic detector methods based on crowd sourced trajectories and street-view imagery to reveal the multidimensional effects of built environment factors (road connectivity, lighting infrastructure, population density, etc.) on jogging activities. Yang et al. (32) employed GPS tracking data to decipher the spatial patterns of residents’ jogging/running activities within diverse typologies of green spaces, validating the effectiveness of big data methods in health geography research.

## 3 Methodology

### 3.1 Data collection and processing

This section details the data collection and processing procedures for this study. Parks represent the most common and representative form of urban green space, widely distributed across different administrative districts and capable of reflecting typical scenarios for PA among urban residents. Therefore, this study focuses on parks in Zhengzhou City, Henan Province as the research subjects for urban green spaces. Six typical parks were selected to ensure representativeness across different administrative districts, size categories, and functional types. The selection criteria included: (1) distribution across six major administrative districts to capture spatial diversity; (2) variation in park size (ranging from medium to large-scale parks); (3) different primary functions (recreational, fitness-oriented, and scenic); and (4) accessibility to diverse demographic groups. The selected parks are: Jinshui People’s Park (central urban area, large-scale recreational park), Zhongyuan Bishagang Park (urban core, medium-scale fitness park), Erqi Rose Park (urban district, medium-scale recreational park), Huiji Yellow River Scenic Area (peripheral area, large-scale scenic park), Guancheng Zijingshan Park (urban district, medium-scale recreational park), and Shangjie Jiyuan Lake Park (peripheral area, medium-scale scenic park). This purposive sampling strategy was designed to capture variation across park types rather than to support formal population-level generalization beyond the selected cases. Data collection and processing were conducted through a combination of field surveys, questionnaires, and VGI data, laying the foundation for subsequent spatiotemporal characteristic analysis and exploration of associated factors.

#### 3.1.1 Data collection methods

Data collection was primarily completed through field surveys conducted during the summer of 2023 (June–August 2023), covering both weekdays and weekends. Time periods included morning (6:00–9:00), midday (11:00–14:00), and evening (17:00–20:00). Research teams of 3–5 members used standardized observation forms to record residents’ age, gender, activity type, intensity, location, and duration. Observations were non-participatory to avoid influencing resident behavior. Multiple observation points were established in each park (e.g., entrance plaza, waterfront promenade, fitness area), with continuous observation at each point lasting 30–60 min. To supplement subjective data, questionnaires were randomly distributed during the same period (summer 2023), yielding 345 valid responses that inquired about activity preferences and relevant environmental and perceptual conditions (e.g., perceived green space quality, sense of safety). The questionnaire items were developed based on existing literature on green space perception and PA behavior, covering five key dimensions: perceived green quality, facility convenience, sense of security, crowding, and noise level. Sense of security was operationalized multidimensionally with subscales for personal safety (e.g., fear of crime or harassment, 3 items), facility safety (e.g., lighting and maintenance quality, 3 items), and social safety (e.g., comfort in social interactions, 2 items), drawing from environmental psychology distinctions (33).

Each dimension was measured using multiple items on a 5-point Likert scale. Prior to the formal survey, a pilot study with 30 participants was conducted to assess item clarity and relevance. Based on pilot feedback, minor wording adjustments were made to improve comprehensibility. Construct validity was evaluated through factor analysis, and internal consistency was assessed using Cronbach’s alpha coefficients. To ensure data reliability, team training was conducted prior to the survey, and collected data underwent independent verification by two individuals. In this study, VGI refers to two complementary forms of user-contributed spatial data: (1) systematic behavioral observation records produced by the field research teams during structured site surveys, in which each of the 18,650 valid records documents one activity instance observed at a specific functional zone (e.g., fitness area, leisure plaza, waterfront promenade) within one of the six study parks. Each record captures the observed individual’s apparent age group, gender, activity type, activity intensity category, time period (morning/midday/evening), and zone-level location. Observers conducted non-participatory walkthroughs of pre-defined functional zones at scheduled time windows; the zone-level spatial coordinates were recorded using GPS-assisted positioning during field surveys. These records were subsequently mapped in GIS by functional zone to characterize the spatial distribution of PA across park areas; and (2) park boundary shape files and facility location data sourced from OpenStreetMap (updated 2023), which were used to provide the spatial reference framework and were cross-validated with on-site GPS surveys. Together, these spatial data constitute the GIS/VGI component of the multi-source dataset. Observation locations were recorded by functional zone (e.g., entrance plaza, waterfront promenade, fitness area) using GPS-assisted positioning during field surveys. Zone-level aggregated counts were subsequently mapped in GIS to characterize the spatial distribution of PA across park areas. The study generated 345 statistical data tables covering 26 PA types (e.g., walking, jogging, square dancing, sitting quietly, chatting, and playing board/card games).

Data collation results are shown in Table 1, yielding 18,650 valid records. Among these, Jinshui People’s Park contributed the largest dataset with 4,680 records, accounting for 25% of the total, reflecting its high usage frequency and representativeness.

**Table 1:** Data collection overview.

Administrative district	Green space name	Number of records	Percentage (%)
Jinshui district	Jinshui People’s Park	4,680	25
Zhongyuan district	Zhongyuan Bishagang Park	2,795	15
Erqi district	Erqi Rose Park	2,795	15
Huiji district	Huiji Yellow River Scenic Area	2,795	15
Guancheng district	Guancheng Zijingshan Park	2,795	15
Shangjie district	Shangjie Jiyuan Lake Park	2,790	15
Total	–	18,650	100

Note: The study collected 18,650 observational activity records and 345 questionnaire responses. The observational and GIS/VGI data are used to describe spatiotemporal activity patterns across the six parks, whereas the questionnaire data provide the respondent-level basis for analyzing associations involving perceived environmental conditions. These two data sources therefore serve complementary but distinct analytical purposes in the manuscript.

For the descriptive analyses, PA frequency refers to the number of valid activity records observed in each park under the standardized survey schedule. PA intensity was coded for each record as 1 = light, 2 = moderate, and 3 = vigorous according to the MET-based categories above. PA duration was recorded in minutes for each observed activity record. Questionnaire-based perception variables (X6–X12) were scored at the respondent level and used in the individual-level regression analyses reported later in the manuscript.

#### 3.1.2 Data processing and preliminary statistics

Spatial and environmental data were collected to temporally align with the behavioral observation period. Specifically, NDVI (Normalized Difference Vegetation Index) data were derived from Landsat 8 satellite imagery acquired in July 2023, coinciding with the peak summer observation period to ensure temporal consistency between vegetation conditions and observed activities. Park boundary shape files and facility locations (fitness equipment, public restrooms) were verified through on-site GPS surveys conducted simultaneously with field observations (summer 2023) and cross-validated with OpenStreetMap data updated in 2023. Public transportation accessibility data (number of surrounding bus stops within 500 m buffer) were obtained from Zhengzhou Public Transport Corporation’s 2023 route maps, current as of June 2023. This temporal alignment protocol ensures that environmental measurements reflect the same timeframe as behavioral observations, minimizing potential temporal mismatch bias that could arise from rapid urban environmental changes.

Data processing utilized Excel, Python (Pandas library), and ArcGIS software, with steps including:

1. Cleaning: Removed missing values and outlier records (e.g., activity duration <1 min), achieving a final valid data rate of 95%.
2. Categorization: Age groups: children (<18 years), young adults (18–35 years), middle-aged adults (36–59 years), older adults (≥60 years); Gender: male/female; Activity intensity graded by METs (metabolic equivalents): light (<3 METs, including sitting quietly, standing, chatting, board games, photography), moderate (3–6 METs, including walking, square dancing, brisk walking), vigorous (>6 METs, including jogging, running, equipment-based exercise). The MET values were assigned based on the Compendium of Physical Activities (34), and activity-specific energy expenditure standards commonly used in PA research.
3. Statistical analysis: Calculated frequencies, proportions, and means. Preliminary results indicate higher activity frequency on weekends than weekdays (approximately 15% difference), with middle-aged and older adult groups accounting for over 60% of participants and male participants dominating (55–70%). These findings lay the foundation for subsequent spatiotemporal characteristic and influencing factor analyses.

Subjective perception factors were quantified using a Likert scale (1–5 point rating) in the questionnaire survey. A score of 1 indicated “strongly disagree” or “very dissatisfied,” while 5 signified “strongly agree” or “very satisfied.” For the crowding and noise items, higher scores indicate greater perceived crowding and greater perceived noise disturbance. The questionnaire yielded 345 valid responses covering perceived green quality, facility accessibility, perceived safety, crowding, and noise levels. Perceived safety subscales showed strong reliability (personal: *α* = 0.82; facility: α = 0.85; social: α = 0.78) and were averaged for analysis, with composites used in regressions to capture multidimensional effects. Scale reliability was assessed using Cronbach’s alpha coefficients to evaluate internal consistency (threshold >0.7 indicating reliability). Factor analysis employed principal component analysis combined with maximum variance rotation to extract factors and calculate cumulative variance explained, verifying variable independence and structural validity. These subjective scores were incorporated as continuous variables into regression models, analyzed alongside objective variables to examine dual-perspective impacts.

### 3.2 Spatiotemporal analysis procedures

To examine the temporal characteristics of PA, this study computed observed activity frequencies by time period (morning, midday, evening), day type (weekday vs. weekend), age group, gender, and activity intensity using the field observation dataset. Chi-square tests were applied to assess whether temporal distributions differed significantly by gender. To examine the spatial characteristics of PA, this study aggregated observation records by functional zone within each park and mapped the dominant activity category per zone in GIS, categorized by age group, gender, and activity intensity. The resulting spatiotemporal patterns are reported in sections 4.1 and 4.2.

### 3.3 Theoretical framework and exploratory expectations

This study adopts an integrated theoretical framework combining the Social Ecological Model (SEM) and Environmental Stress–Restoration Theory as interpretive lenses to guide variable selection and result interpretation, explicitly framing the ensuing statements as exploratory expectations rather than confirmatory hypotheses. The SEM posits that physical activity behavior emerges from multi-level interactions among individual characteristics, social contexts, and environmental attributes. In this study, this framework suggests that objective environmental features (structural opportunities) and subjective perceptions (psychological interpretations) may be associated with observed physical activity patterns.

Environmental Stress–Restoration Theory complements the SEM by offering a framework for interpreting how environmental stressors (e.g., noise, crowding) and restorative elements (e.g., greenery, perceived safety) may relate to behavioral engagement. Stressors deplete cognitive resources and reduce activity endurance, while restorative features enhance psychological comfort and prolong engagement. Within this dual-framework perspective, we outline the following exploratory expectations about perceived environmental associations with physical activity outcomes, which serve as interpretive guides rather than statistical tests.

Exploratory expectation 1 (Structural Opportunity Associations): Larger park area (X_1_), higher accessibility (X_5_, measured by surrounding bus stops), and better facility provision (X_3_, X_4_) are expected to be positively associated with PA frequency and duration, as they may expand opportunity structures for diverse activities.

Exploratory expectation 2 (Environmental Quality Associations): Higher vegetation coverage (X_2_) and perceived green quality (X_6_) are expected to be positively associated with PA engagement through environmental attractiveness and restorative potential.

Exploratory expectation 3 (Perceived Safety Associations): Multidimensional perceived safety—personal safety (X_8_, fear reduction), facility safety (X_9_, lighting/maintenance), and social safety (X_10_, interpersonal comfort)—is expected to be positively associated with PA frequency, intensity, and duration, with facility safety expected to show the clearest association with evening and prolonged activities due to lighting infrastructure.

Exploratory expectation 4 (Environmental Stressor Associations): Perceived crowding (X_11_) and noise (X_12_) are expected to be negatively associated with PA frequency, intensity, and duration because environmental stress may disrupt restorative experiences and sustained engagement.

Exploratory expectation 5 (Modality-Specific Association Patterns): The associations of environmental factors may vary across PA dimensions: accessibility and facility provision may be more closely related to frequency (visit initiation), safety and comfort factors may be more closely related to intensity (activity vigor), and convenience facilities (restrooms) and restorative features may be more closely related to duration (sustained engagement).

### 3.4 Model construction

The modeling strategy of this study follows the evidentiary hierarchy stated above. Descriptive spatiotemporal analyses based on the multi-source observational dataset and respondent-level questionnaire regressions (*n* = 345) provide the empirical basis for interpretation. To clarify the analytical role of each dataset, this study distinguishes between two levels of evidence. The field observation and GIS/VGI data are used primarily to document the spatiotemporal characteristics of park-based PA. The questionnaire dataset (*n* = 345) provides the analytical basis for examining associations involving perceived environmental conditions at the individual level.

#### 3.4.1 Multiple linear regression model

Before conducting regression analysis, pairwise correlations and variance inflation factor (VIF) diagnostics were examined to screen for serious multicollinearity among predictors. The questionnaire-based regression models use self-reported PA outcomes as dependent variables: PA frequency (visits per week), PA intensity (self-rated activity vigor on a 1–5 scale), and PA duration (average minutes per visit). Independent variables include the seven perception-based measures: perceived green quality (X_6_), facility convenience (X_7_), personal safety (X_8_), facility safety (X_9_), social safety (X_10_), crowding (X_11_), and noise (X_12_). Control variables include respondent age, gender, and park visited. The basic model form of multiple linear regression is shown in Equation 1:


$$y_{i}=\beta_{0}+\sum_{k=1}^{p}\;\beta_{k}X_{\mathrm{ki}}+\varepsilon_{i},\varepsilon_{i}∼N(0,\sigma^{2})$$ (1)

Where, $y_{i}$ denotes the dependent variable for the *i*-th respondent, $\beta_{0}$ is the intercept, $\beta_{k}$ denotes the regression coefficient for predictor *k*, and $X_{\mathrm{ki}}$ denotes the corresponding independent variable. $\varepsilon_{i}$ represents the random error term.

Model estimation employs the OLS method, minimizing the sum of squared residuals as shown in Equation 2:


$$\min\sum_{i=1}^{n}\in_{i}^{2}=\sum_{i=1}^{n}{\left(y_{i}-{\hat{y}}_{i}\right)}^{2}$$ (2)

Where ${\hat{y}}_{i}={\hat{\beta }}_{0}+\sum_{k=1}^{p}\;{\hat{\beta }}_{k}X_{\mathrm{ki}}$ is the predicted value. As shown in Equation 3, Model goodness-of-fit is assessed using adjusted *R*^2^:


$$R^{2}=1-\frac{\sum \epsilon_{i}^{2}/(n-p-1)}{\sum {\left(y_{i}-\bar{y}\right)}^{2}/(n-1)}$$ (3)

Standardized coefficients, standard errors, t-statistics, and *p*-values are reported to describe the direction and relative strength of the observed respondent-level associations. Although PA intensity is measured on a five-point self-rated scale, the use of OLS for this outcome is consistent with established practice in quantitative social science research. Simulation studies and analytical comparisons have shown that when the number of response categories is five or more and the distribution is not severely skewed, OLS regression and ordinal regression (e.g., proportional odds models) produce substantively equivalent results in terms of the direction, relative magnitude, and statistical significance of associations. In the present sample, the PA intensity ratings are approximately symmetrically distributed around the midpoint, and preliminary sensitivity checks confirmed that the pattern of significant associations was consistent across OLS and ordinal estimation. OLS is therefore retained for interpretive simplicity and comparability with prior literature using the same outcome scaling. PA frequency (visits per week) and PA duration (average minutes per visit) are treated as approximately continuous outcomes, which is standard in park-based PA research.

Regarding the variable framework, X_1_–X_5_ represent park-level objective environmental attributes (area, NDVI, facility count, facility type, and public transit accessibility). These variables are park-level constants: each respondent is associated with a single park, so X_1_–X_5_ are perfectly collinear with the park fixed effects already included in the models. Including both park fixed effects and park-level covariates would result in multicollinearity and prevent identification. X_1_–X_5_ therefore serve the spatiotemporal descriptive track (section 3.3 and 4.1–4.2) rather than the individual-level regression track, and their exclusion from the questionnaire-based models is a structural consequence of the park fixed-effect design.

## 4 Results

### 4.1 Temporal characteristics of PA

**Figure 1:**
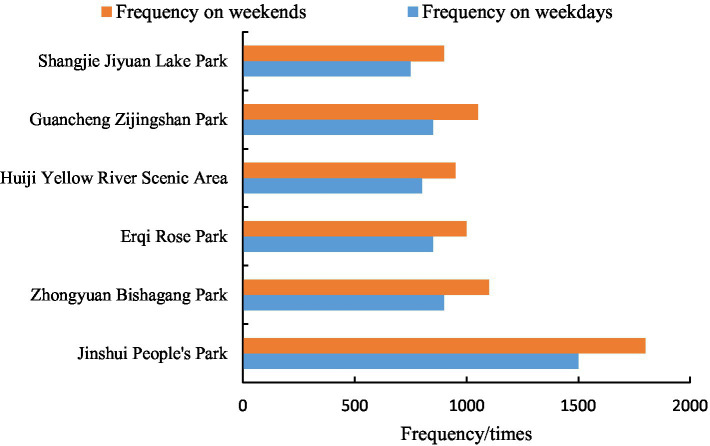
Frequency statistics of activities in urban green spaces.

Figure 1 illustrates the overall frequency of PA observed at six representative urban green spaces in Zhengzhou across different time periods throughout the week. The observed data indicate that overall PA frequency is generally higher on weekends than on weekdays (approximately 15% difference), reflecting temporal patterns likely associated with work-rest cycles in the urban population. This temporal variation suggests that park usage in the observed sample responds to community-wide leisure time availability. The pattern highlights the connection between observed activity and societal daily rhythms. Furthermore, although weekend overall activity frequency exceeds weekdays, the gap is relatively moderate, suggesting these urban green spaces maintain consistent activity levels throughout the week. This sustained weekday usage pattern may reflect contributions from various subpopulations utilizing different time windows (e.g., morning and evening peaks, midday visitors from nearby workplaces).

#### 4.1.1 Temporal characteristics of age distribution among users of urban green spaces

Statistical analysis of collected data reveals temporal patterns in the age composition of observed park users across six representative urban green spaces in Zhengzhou. As shown in Table 2, middle-aged and older adult individuals constitute the dominant demographic observed, accounting for over 60% of total observed activity instances across all six parks. This overall pattern indicates that these parks primarily serve middle-aged and older adult populations, though the specific age composition varies across different sites. Specifically, observed older adults activity frequency showed minimal temporal fluctuation. Notably, Jinshui People’s Park and Guancheng Zijingshan Park recorded higher older adult presence, particularly during midday observations. In the remaining four parks, observed older adults activity levels remained relatively consistent between midday and evening periods. This stability may reflect park-specific characteristics such as shaded areas and scheduled community programming. Observed child presence peaked during midday, with the highest frequencies recorded at Jinshui People’s Park and Huiji Yellow River Scenic Area, followed by Erqi Rose Park and Guancheng Zijingshan Park, while Shangjie Jiyuan Lake Park showed the lowest child activity. Observed young adult presence was relatively low, peaking at midday, with the highest frequencies at Zhongyuan Bishagang Park, followed by Jinshui People’s Park, Erqi Rose Park, and Shangjie Jiyuan Lake Park, and lowest at Huiji Yellow River Scenic Area. Overall, observed activity frequency across all age groups was lowest in early morning and peaked during midday, with older adult presence remaining comparable between midday and evening periods.

**Table 2:** Age statistics of people engaged in urban green space activities.

Green space name	Age group	Morning frequency (times)	Midday frequency (times)	Evening frequency (times)
Jinshui People’s Park	Children (<18 years old)	220	350	250
	Young adults (18–35 years old)	170	300	200
	Middle-aged adults (36–59 years old)	320	450	350
	Older adults (≥60 years old)	450	600	550
Zhongyuan Bishagang Park	Children (<18 years old)	200	320	230
	Young adults (18–35 years old)	220	400	250
	Middle-aged adults (36–59 years old)	300	420	320
	Older adults (≥60 years old)	400	550	530
Erqi Rose Park	Children (<18 years old)	190	310	220
	Young adults (18–35 years old)	160	280	190
	Middle-aged adults (36–59 years old)	310	430	330
	Older adults (≥60 years old)	410	560	540
Huiji Yellow River Scenic Area	Children (<18 years old)	210	340	240
	Young adults (18–35 years old)	150	260	180
	Middle-aged adults (36–59 years old)	290	410	310
	Older adults (≥60 years old)	390	540	520
Guancheng Zijingshan Park	Children (<18 years old)	200	330	230
	Young adults (18–35 years old)	180	290	200
	Middle-aged adults (36–59 years old)	320	440	340
	Older adults (≥60 years old)	460	590	570
Shangjie Jiyuan Lake Park	Children (<18 years old)	180	300	210
	Young adults (18–35 years old)	160	270	190
	Middle-aged adults (36–59 years old)	280	400	300
	Older adults (≥60 years old)	380	530	510

#### 4.1.2 Temporal characteristics of gender distribution among users of urban green spaces

Table 3 reveals temporal patterns in the gender composition of observed park users across six representative urban green spaces in Zhengzhou. As shown in Table 3, male presence predominates in observed activity instances, with higher male-to-female ratios observed at Jinshui People’s Park, Zhongyuan Bishagang Park, and Erqi Rose Park. Specifically, on weekdays, observed male presence peaks during morning hours at Jinshui People’s Park, Guancheng Zijingshan Park, and Zhongyuan Bishagang Park. Huiji Yellow River Scenic Area exhibits a distinct temporal pattern, with male presence peaking during evening hours. Observed female presence on weekdays shows a gradual increase from morning, peaking during midday, then declining thereafter. Comparing the temporal distributions reveals that observed male peaks concentrate in morning periods, while observed female peaks occur during midday. The gender difference in observed activity frequency is minimal in morning periods, while female presence slightly exceeds male presence in evening periods.

**Table 3:** Gender statistics of people engaged in urban green space activities.

Green space name	Gender	Morning frequency (times)	Midday frequency (times)	Evening frequency (times)
Jinshui People’s Park	Male	800	700	600
	Female	500	650	550
Zhongyuan Bishagang Park	Male	750	650	550
	Female	450	600	500
Erqi Rose Park	Male	780	680	580
	Female	480	630	530
Huiji Yellow River Scenic Area	Male	600	700	800
	Female	400	550	600
Guancheng Zijingshan Park	Male	770	670	570
	Female	470	620	520
Shangjie Jiyuan Lake Park	Male	700	600	500
	Female	420	570	470

Furthermore, this study conducted a chi-square test using the combined observations across all six parks. The results show a significant association between gender and time period (χ^2^ = 138.42, d*f* = 2, *p* < 0.001), indicating that the temporal distribution of activities differs systematically between males and females. Additional chi-square tests performed for each park separately also show significant gender–time associations (*p* < 0.05 for all six parks), confirming that these temporal differences are consistent across locations.

#### 4.1.3 Temporal characteristics of PA intensity distribution

Table 4 presents the classification and statistics of activities at different intensities (summarized by park). Results indicate that light-intensity activities dominate in green spaces, accounting for approximately 50% of total activity frequency. This may stem from parks’ suitability for leisure and relaxation. Moderate- and vigorous-intensity PA exhibits lower proportions at 30 and 20% respectively, aligning with urban residents’ preference for leisurely rather than strenuous daily activities. Regarding temporal patterns, vigorous-intensity PA peaks in the morning (likely due to morning exercise routines), light activity dominates at midday (lunchtime leisure), and moderate activity increases in the evening (after-work strolls). Activity intensity distributions vary across green spaces: for instance, vigorous-intensity PA at Jinshui People’s Park is concentrated in the morning, while moderate activity at Zhongyuan Bishagang Park peaks in the evening.

**Table 4:** Frequency statistics of physical activities at different intensities among people engaged in urban green space activities.

Green space name	Intensity level	Morning frequency (times)	Midday frequency (times)	Evening frequency (times)
Jinshui People’s Park	Light	300	600	400
	Moderate	200	300	280
	Vigorous	240	160	120
Zhongyuan Bishagang Park	Light	280	580	380
	Moderate	190	290	300
	Vigorous	220	150	110
Erqi Rose Park	Light	290	590	390
	Moderate	195	295	285
	Vigorous	230	155	115
Huiji Yellow River Scenic Area	Light	270	570	370
	Moderate	185	285	275
	Vigorous	210	145	105
Guancheng Zijingshan Park	Light	295	595	395
	Moderate	200	300	290
	Vigorous	235	160	120
Shangjie Jiyuan Lake Park	Light	260	560	360
	Moderate	180	280	270
	Vigorous	200	140	100

### 4.2 Spatial characteristics of PA

Different groups exhibit varying time preferences for activities in green spaces. Understanding activity timing alone is insufficient for comprehensively assessing green space use. Therefore, this study further analyses the spatial characteristics of PA, including age, gender, and intensity patterns. Overall spatial distribution reflects the vitality of green space sites—the locations with the highest frequency of human presence. Through zone-level aggregation and GIS mapping of observational data, this study reveals spatial pattern differences across six typical green spaces in Zhengzhou.

#### 4.2.1 Spatial distribution characteristics by age group

**Figure 2:**
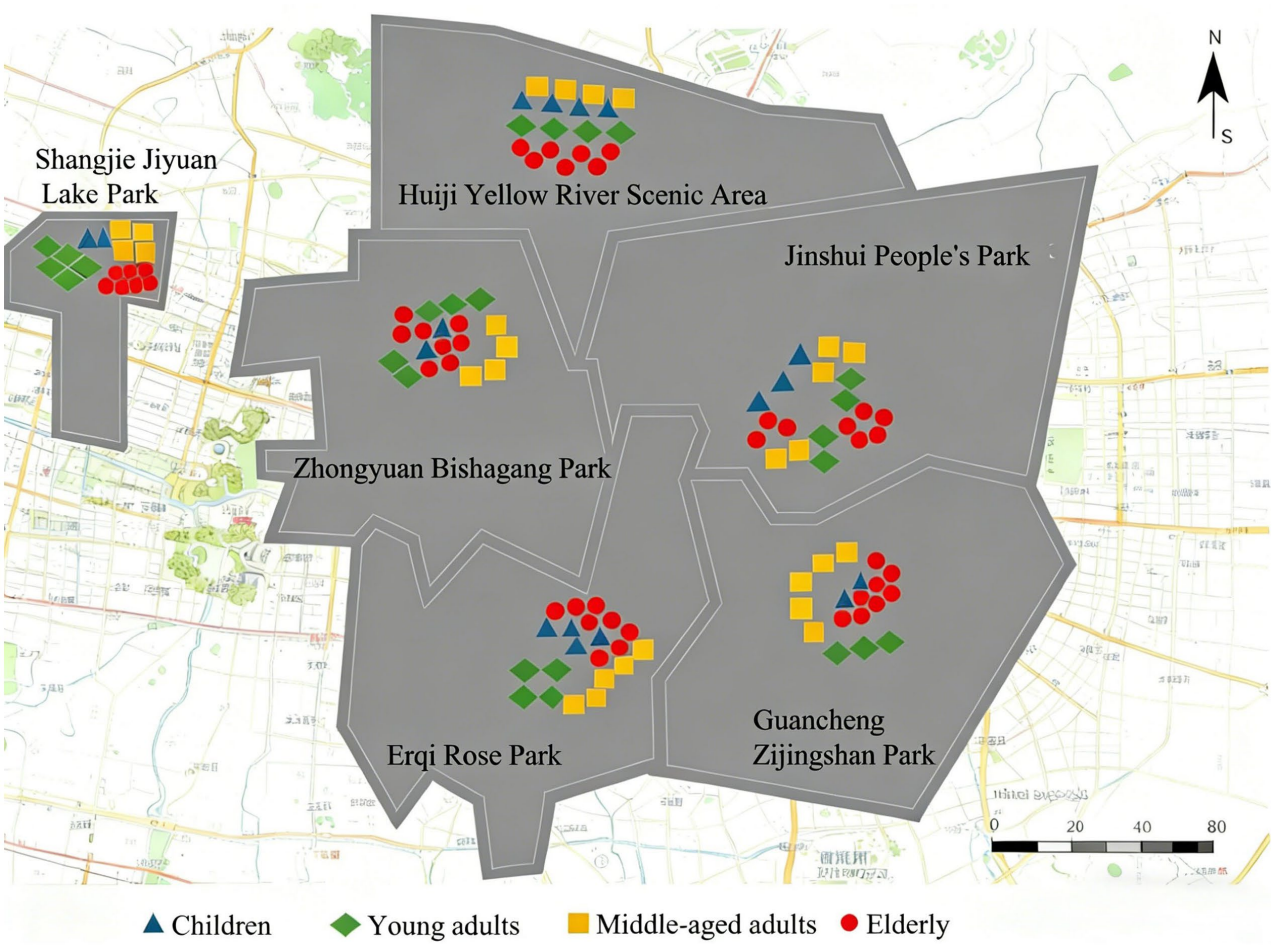
Spatial distribution of observed PA records across different age groups (aggregated by functional zone).

The spatial distribution patterns of PA among different age groups are shown in Figure 2. As illustrated, observed older adult presence concentrates in leisure zones and plazas, with moderate activities like square dancing or morning exercises, as well as light activities such as walking and socializing. Observed middle-aged individual presence predominantly occurs in leisure zones and greenways, with moderate activities like brisk walking or family strolls. Young adult observations are predominantly recorded in fitness zones, with vigorous-intensity activities like running or equipment workouts. Children are mainly observed in recreational zones, participating in light activities such as playing games or walking. This spatial pattern reflects the functional differentiation and age-specific utilization patterns of green spaces observed in this study.

#### 4.2.2 Spatial characteristics of PA gender distribution

**Figure 3:**
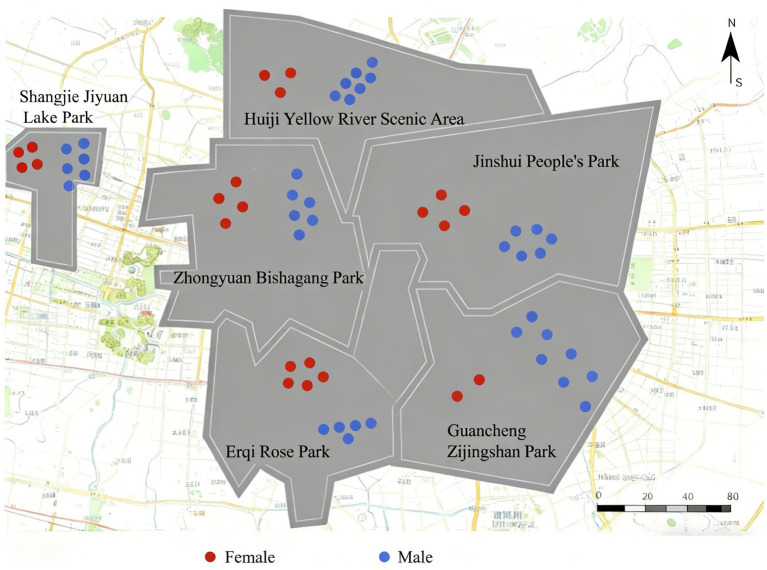
Spatial distribution of observed PA records by gender (aggregated by functional zone).

Figure 3 reveals the spatial distribution patterns of observed PA across different gender groups. As shown in Figure 3, observed male presence concentrates in fitness zones, where vigorous-intensity activities such as weight training or running predominate; observed female presence exhibits broader distribution across leisure zones, where moderate-to-light intensity activities like walking or social interaction predominate. Both gender groups are present across all parks, though spatial distribution patterns differ: male presence concentrates in peripheral fitness zones; female presence concentrates in central leisure zones. Overall, observed male activity instances outnumber female instances across all six parks. This gendered spatial segregation pattern may reflect the combined influence of differentiated activity preferences, accessibility patterns, and socio-cultural factors affecting park usage. Within the SEM framework, such patterns are consistent with gendered social norms and environmental perceptions. Previous research documents gender differences in environmental sensitivity and activity selection at the individual level; our observations capture the spatial manifestation of these processes in visible usage patterns. However, without individual-level data on motivations and perceptions, we cannot directly attribute these observed patterns to specific individual-level mechanisms.

#### 4.2.3 Spatial characteristics of PA intensity distribution

**Figure 4:**
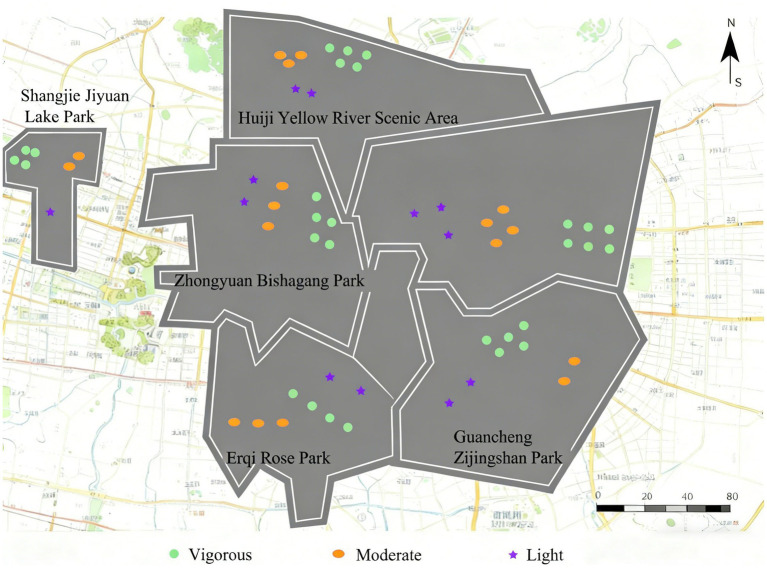
Spatial distribution of observed PA records by intensity category (aggregated by functional zone).

The spatial distribution of physical activities at different intensities is shown in Figure 4. To visually illustrate variations, the study employed a GIS platform to conduct kernel density analysis on the observational data, calculating point feature densities and generating spatial distribution maps. The spatial distribution map categorizes activity density into three levels—light, moderate, and heavy—using the natural breakpoint method for classification. Results indicate consistent patterns across green spaces in PA intensity distribution. Specifically: Light PA (including standing, photography, sitting quietly, chatting, board games, singing, etc.) is primarily concentrated in recreational zones and waterfront areas. Moderate PA (including brisk walking and square dancing) was predominantly distributed along greenways and plazas. Vigorous-intensity PA (including running and equipment-based exercise) was concentrated in fitness zones. This spatial differentiation pattern reveals the universal environmental preferences for physical activities of varying intensities.

### 4.3 Descriptive statistics and measurement reliability of perception variables

Table 5 summarizes the descriptive statistics and reliability indicators of the subjective perception variables. The reliability test of the scale shows that the overall Cronbach’s *α* is 0.82 (>0.7, indicating good internal consistency). The α values of each sub-scale are as follows: perceived green quality 0.78, perceived facility convenience 0.81, perceived crowding 0.76, and perceived noise level 0.80. Perceived safety subscales: personal (X_8_, α = 0.82), facility (X_9_, α = 0.85), social (X_10_, α = 0.78). Factor analysis (principal component analysis + varimax rotation) extracted five factors, with a cumulative variance explanation rate of 72.5%, confirming the independence of the variables. As shown in Table 5, the mean of X_6_ is 3.8 (SD = 0.9), the mean of X_7_ is 3.5 (Standard Deviation (SD) = 1.0), the mean of X_8_ is 3.7 (SD = 0.7), X_9_ is 3.5 (SD = 0.9), X_10_ is 3.6 (SD = 0.8), the mean of X_11_ is 2.4 (SD = 1.1), with higher values indicating greater perceived crowding, and the mean of X_12_ is 2.7 (SD = 1.0), with higher values indicating greater perceived noise disturbance.

**Table 5:** Descriptive statistical results.

Variable	Mean	SD	Cronbach’s α
X_6_	3.8	0.9	0.78
X_7_	3.5	1	0.81
X_8_	3.7	0.7	0.82
X_9_	3.5	0.9	0.85
X_10_	3.6	0.8	0.78
X_11_	2.4	1.1	0.76
X_12_	2.7	1	0.8
Overall	-	-	0.82

### 4.4 Individual-level questionnaire analyses

Although the observational dataset contains a large number of activity records, these records are event-based observations rather than independent respondent-level cases. They do not include respondent identifiers, full socio-demographic covariates, or perception-based measures that would support meaningful individual-level behavioral modeling. In addition, many observations are clustered within the same parks and observation periods, so treating them as an individual-level regression sample would risk pseudo-replication. For this reason, the individual-level analyses in this study are based on the questionnaire dataset (*n* = 345), which provides respondent-level perception variables suitable for examining behavioral associations.

#### 4.4.1 Individual-level regression design

The individual-level models use self-reported PA measures as dependent variables: (1) PA frequency (visits per week), (2) PA intensity (self-rated activity vigor on a 1–5 scale), and (3) PA duration (average minutes per visit). Independent variables include the seven perception-based measures: perceived green quality (X_6_), facility convenience (X_7_), personal safety (X_8_), facility safety (X_9_), social safety (X_10_), crowding (X_11_), and noise (X_12_). Control variables include respondent age, gender, and park visited. These models constitute the primary analytical basis for evaluating perception-related associations in this study.

#### 4.4.2 Individual-level regression results

Table 6 presents the main individual-level regression results for PA frequency and intensity. Because these models are estimated on the 345 questionnaire respondents and include respondent-level perception measures with basic controls, they provide the primary empirical basis for interpreting associations involving perceived environmental conditions in this study. The frequency model (*R*^2^ = 0.218, *F* = 11.34, *p* < 0.001) shows that facility convenience and personal safety are positively associated with visit frequency, whereas perceived noise and crowding show significant negative associations. The intensity model (*R*^2^ = 0.195, *F* = 9.87, *p* < 0.001) similarly indicates positive associations for facility convenience and social safety, with negative associations for noise and crowding.

**Table 6:** Individual-level regression results for PA frequency and intensity (*n* = 345).

Variable	PA frequency			PA intensity		
	β	SE	*t*-value	β	SE	*t*-value
Intercept	1.312	0.298	4.40***	2.087	0.285	7.32***
X_6_ (green quality)	0.098	0.054	1.81*	0.072	0.052	1.38
X_7_ (facility convenience)	0.156	0.050	3.12**	0.142	0.048	2.96**
X_8_ (personal safety)	0.118	0.047	2.51**	0.078	0.045	1.73*
X_9_ (facility safety)	0.076	0.049	1.55	0.065	0.047	1.38
X_10_ (social safety)	0.058	0.053	1.09	0.125	0.051	2.45**
X_11_ (crowding)	−0.132	0.046	−2.87**	−0.154	0.044	−3.50***
X_12_ (noise)	−0.167	0.048	−3.48***	−0.189	0.046	−4.11***
Age (control)	0.042	0.036	1.17	−0.028	0.034	−0.82
Gender (control)	0.054	0.085	0.64	0.098	0.081	1.21
R^2^	0.218			0.195		
Adj. R^2^	0.195			0.171		
F-statistic	11.34*			9.87*		

***,**,*Represent significance at 1, 5, and 10% levels, respectively. β represents standardized coefficients. Park fixed effects included but not shown.

Table 7 presents the main individual-level results for PA duration. The model (*R*^2^ = 0.207, *F* = 10.62, *p* < 0.001) shows that perceived green quality, facility convenience, personal safety, and facility safety are positively associated with activity duration, whereas perceived noise and crowding show negative associations.

**Table 7:** Individual-level regression results for PA duration (*n* = 345).

Variable	β	SE	*t*-value	*p*-value
Intercept	14.876	3.312	4.49	0.000***
X_6_ (green quality)	0.145	0.053	2.74	0.007**
X_7_ (facility convenience)	0.128	0.049	2.61	0.010**
X_8_ (personal safety)	0.095	0.046	2.07	0.039**
X_9_ (facility safety)	0.138	0.048	2.88	0.004**
X_10_ (social safety)	0.062	0.052	1.19	0.235
X_11_ (crowding)	−0.108	0.045	−2.40	0.017**
X_12_ (noise)	−0.176	0.047	−3.74	0.000***
Age (control)	0.078	0.035	2.23	0.027**
Gender (control)	−0.041	0.084	−0.49	0.626
R^2^	0.207			
Adj. R^2^	0.184			
F-statistic	10.62*			

***,**,*Represent significance at 1, 5, and 10% levels, respectively. β represents standardized coefficients. Park fixed effects included but not shown.

## 5 Discussion

The following discussion interprets the findings from the two complementary analytical tracks of this study. Section 5.1 addresses the spatiotemporal descriptive findings based on the observational dataset, and section 5.2 addresses the individual-level perception findings based on the questionnaire dataset. These tracks are discussed separately because they employ different data, address different research questions, and support different types of interpretive claims.

### 5.1 Interpretation of spatiotemporal descriptive findings

The descriptive results indicate clear temporal concentration in park-based PA across the six study parks, with higher overall participation on weekends and recurrent peaks during midday and evening periods. These patterns are consistent with daily work-rest rhythms and suggest that park use is closely tied to residents’ available leisure time. The strong participation of middle-aged and older adults further indicates that urban parks function as important routine activity spaces for these groups in Zhengzhou.

The spatial analysis also shows marked functional differentiation within parks. Leisure, fitness, and recreation zones emerged as the main activity hotspots, while different activity intensities displayed distinct spatial preferences. Light activities were concentrated in leisure and waterfront spaces, moderate activities were common along greenways and plazas, and vigorous activities clustered in fitness zones. Together, these findings suggest that internal park layout and functional zoning are closely related to how users distribute their activities across space.

Gender and age differences add further nuance to these patterns. Male users were more concentrated in fitness-oriented areas, whereas female users were more widely distributed in leisure spaces. Middle-aged and older adults accounted for the largest share of observed users, while children and younger adults showed more time-specific participation. These differences imply that park-based PA is shaped not only by general environmental conditions but also by group-specific needs and activity preferences.

### 5.2 Interpretation of individual-level questionnaire findings

The questionnaire-based regressions provide the main evidence for understanding perception-related associations in this study. Across the models, facility convenience shows a relatively stable positive association with PA frequency, intensity, and duration, suggesting that accessible and usable amenities may support both visit initiation and sustained engagement. Perceived safety also shows positive associations, although the specific dimensions vary by outcome: personal safety is more closely linked to frequency and duration, whereas social safety is more clearly associated with activity intensity.

Environmental stressors show a more consistent pattern. Perceived noise and crowding are negatively associated with multiple PA outcomes, indicating that excessive disturbance may discourage visits, reduce activity intensity, and shorten time spent in parks. Perceived green quality is positively associated with duration, suggesting that attractive and well-maintained green environments may be especially relevant for sustaining longer stays rather than simply increasing visit counts.

Taken together, these respondent-level findings are broadly consistent with existing literature emphasizing convenience, safety, and environmental comfort as important correlates of park-based PA. At the same time, the outcome-specific differences across frequency, intensity, and duration indicate that different dimensions of PA may respond to somewhat different aspects of perceived park quality.

### 5.3 Implications for urban planning

The spatiotemporal patterns identified in this study provide actionable insights for urban planners. The concentration of activity hotspots in leisure, fitness, and recreational zones suggests that functional zoning within parks is effective in attracting users. Planners should consider: (1) ensuring adequate provision of fitness equipment and facilities in designated fitness zones to support vigorous activities; (2) maintaining well-lit pathways and open spaces in leisure zones to enhance perceived safety, particularly for evening activities; (3) designing age-appropriate facilities, such as playgrounds for children and exercise equipment suitable for older adult users; and (4) strategically locating parks near public transportation hubs to improve accessibility.

The individual-level evidence for the negative associations of noise and crowding, together with the broader descriptive findings on park use patterns, suggests that park management should consider: (1) implementing noise reduction measures, such as vegetation buffers or sound barriers near high-traffic areas; (2) improving circulation design and spatial zoning to reduce perceived crowding; and (3) creating quieter subspaces within parks for activities requiring lower levels of environmental disturbance.

### 5.4 Limitations and future research

Several limitations should be acknowledged. First, the study is based on six purposively selected parks in Zhengzhou, so the findings should be interpreted as context-specific rather than broadly generalizable to all urban parks. Second, the questionnaire-based analyses are cross-sectional and therefore cannot support causal inference; the observed associations may reflect reciprocal relationships or omitted contextual factors. Third, the questionnaire outcomes and perceived environmental variables rely on self-report, which may be affected by recall bias or subjective response tendencies. Fourth, observational coding of activity intensity, although useful for large-scale field recording, is less precise than device-based measurement. Finally, the current design does not fully integrate intra-park spatial heterogeneity with respondent-level perceptions, and future research would benefit from broader park samples, richer respondent covariates, and multilevel or spatially explicit designs.

## 6 Conclusion

This study examines park-based physical activity in six urban parks in Zhengzhou through two complementary analytical tracks: observational data documenting spatiotemporal patterns, and questionnaire-based individual-level analyses examining perceived environmental associations with PA outcomes. This study provides a multi-source description of the spatiotemporal characteristics of residents’ physical activity in six urban parks in Zhengzhou. The results show clear temporal concentration in midday and evening periods, substantial participation by middle-aged and older adults, and spatial clustering of activities in leisure, fitness, and recreation zones.

The questionnaire-based individual-level analyses further suggest that perceived safety, facility convenience, environmental quality, noise, and crowding are associated with PA-related outcomes at the respondent level. These findings strengthen the paper’s contribution as a study that combines spatiotemporal description with individual-level analysis of perceived environmental correlates.

Overall, the study provides evidence that urban park design and management should pay attention not only to functional zoning and activity-supportive facilities, but also to perceived safety, crowding, and environmental comfort. Future research should expand the park sample and adopt stronger multilevel or spatially explicit designs to further examine how park environments shape physical activity behavior.

## Data Availability

The datasets presented in this study can be found in Figshare: https://doi.org/10.6084/m9.figshare.30165223.
